# 

**Published:** 1873-07

**Authors:** 


JULY, 1873.

### THE AMERICAN JOURNAL OF DENTAL SCIENCE,

EDITED BY

F. J. S. GORGAS, M. D., D. D. S.

VOL. 7.—THIRD SERIES.—No. 3.

BALTIMORE:

SNOWDEN & COWMAN.

LONDON:

TRUBNER & CO., 60 PATERNOSTER ROW.

1873.

$2.50 PER ANNUM.

PAYABLE IN ADVANCE.

